# Evaluating Vitamin D Deficiency-Linked Digestive Issues by Bridging Endocrinology With Gastrointestinal Disorders

**DOI:** 10.7759/cureus.89598

**Published:** 2025-08-08

**Authors:** Wachi Jain, M Khaliq, Abdul Wahab, Mubina Laghari

**Affiliations:** 1 Medicine, Lincoln County Hospital, Lincoln, GBR; 2 Pathology, University of Health Sciences, Lahore, PAK; 3 Internal Medicine, Sargodha Medical College, Sargodha, PAK; 4 Biochemistry, Liaquat University of Medical and Health Sciences, Karachi, PAK

**Keywords:** 25-hydroxyvitamin d2, endocrine system diseases, gastrointestinal disorders, parathyroid hormone, vitamin d deficiency

## Abstract

Background: Vitamin D insufficiency is increasingly recognized as a significant and underlying contributor to a wide range of musculoskeletal disorders, particularly in gastrointestinal (GI) and endocrine health. The study aims to determine the clinical relationship between vitamin D status and the severity of GI symptoms, while also assessing the impact of related endocrine disturbances.

Methods: A cross-sectional study was conducted involving 120 adult patients with GI problems, including constipation, bloating, irritable bowel syndrome (IBS), and dyspepsia in a tertiary care hospital over a six-month duration. Measurement of serum 25-hydroxyvitamin D (25(OH)D), cortisol, and parathyroid hormone (PTH) was obtained. GI signs were documented on validated symptom scales, and correlations were analyzed by Pearson correlation.

Results: Among 120 participants, 81.7% (98) were vitamin D-deficient with an average serum level of 16.8 ng/mL. Patients with a deficiency had significantly more symptoms of IBS and high values of symptom severity scores. The decreased vitamin D was correlated with severe reflux (r = -0.31, p = 0.009), constipation (r = -0.42, p < 0.001), belly pain (r = -0.37, p = 0.003), gas and bloating (r = -0.34, p = 0.005), and bowel incontinence (r = -0.27, p = 0.018). It was also moderately and negatively correlated (r = -0.29, p = 0.012) with the fatty liver index. Furthermore, 65% of individuals had increased levels of cortisol and PTH.

Conclusion: Vitamin D deficiency is common in patients with chronic GI symptoms and correlates with increased symptom severity and endocrine disruptions. Functional GI disorders can be clinically managed by incorporating vitamin D and hormonal testing into regular diagnostics. Identifying and addressing these factors early may help develop effective and personalized treatment approaches.

## Introduction

Vitamin D, long identified for its role in bone health, has recently gained increased interest due to its overall physiological effects, particularly in the areas of gastrointestinal (GI) and endocrine systems [1]. Functional GI disorders (FGIDs), including irritable bowel syndrome (IBS), dyspepsia, and constipation, are frequently observed in clinical practice [2]. Recent studies also indicated that a high percentage of people with persistent GI conditions had vitamin D deficiency, which can affect immune regulation, mucosal integrity, and gut neuromuscular coordination [3]. However, the importance of micronutrient deficiencies in the maintenance and severity of these conditions is not well understood [4].

In addition to lifestyle and other environmental exposures, vitamin D levels are also affected by hormones, including parathyroid hormone (PTH) and cortisol [5]. These endocrine variables are frequently high in individuals with vitamin D deficiency and are associated with impaired digestive function and increased symptoms [6]. Despite the increasing interest in this area, the combined role of vitamin D deficiency and endocrine disruption on digestive complaints, especially in populations with congruent metabolic or lifestyle risk factors, is understudied [7]. Therefore, it limits the identification of modifiable factors that contribute to GI symptoms and delays the development of customized care strategies, which may provide the foundation for designing specific nutritional and hormonal interventions that would optimize the outcomes of chronic GI conditions [8].

The objective of this study is to evaluate the vitamin D deficiency status in individuals with chronic GI symptoms and its relationship with both hormonal markers and the severity of symptoms. It also aims to determine the potential impact of these biological markers on patient management and personalized treatment strategies.

## Materials and methods

A cross-sectional study design was used to examine the relationship between vitamin D deficiency, the severity of GI symptoms, and endocrine disorders. The study was conducted in a tertiary care hospital in the gastroenterology and endocrinology outpatient clinics from September 2023 to November 2023 at Sheikh Zayed Medical Complex Lahore (ref: 331/23). All the participants signed written informed consent to ensure voluntary participation and confidentiality.

A non-probability consecutive sampling technique was used to recruit participants. The sample size was estimated by OpenEpi version 3.0.0 (released 2013, Atlanta, GA, USA) based on a prevalence of 80%, at a 95% confidence interval (Z = 1.96) and a margin of error of 8% [9]. Allowing for 20% non-respondents, the final sample size was 120 participants. Participants aged 18-65 years with features of chronic GI symptoms such as bloating, constipation, IBS, and dyspepsia for at least three months were enrolled. Individuals with known malabsorption syndromes, chronic kidney or liver disease, recent vitamin D intake within the past three months, and pregnancy were excluded from the study.

Patients were not categorized into formal vitamin D sufficient and insufficient groups; however, data were analyzed according to vitamin D status (deficient <20 ng/mL, sufficient 20 ng/mL, or more). Serum vitamin D level was the main exposure, while PTH and cortisol (6 AM) were other related hormonal parameters measured via the enzyme-linked immunosorbent assay (ELISA) method. Age, gender, body mass index (BMI), symptom duration, physical inactivity, and self-reported comorbidities (diabetes mellitus and hypothyroidism) were other variables measured. Routine blood tests to evaluate 25-hydroxyvitamin D (25(OH)D), PTH, and cortisol coupled with the following validated GI symptom questionnaires were used to collect data in this study: Patient-Reported Outcomes Measurement Information System (PROMIS) Scale v1.0-Gastrointestinal Reflux 13a, PROMIS Scale v1.0-Gastrointestinal Constipation 9a, PROMIS Scale v1.0-Gastrointestinal Belly Pain 5a, and PROMIS Scale v1.1-Gastrointestinal Gas and Bloating 13a [10,11]. These PROMIS-GI scales were obtained freely from the official PROMIS repository at www.HealthMeasures.net, which enables open access and validated patient-reported outcome measures [12].

SPSS version 26.0 (released 2019, IBM Corp., Armonk, NY, USA) was used to analyze data. Baseline characteristics were summarized using descriptive statistics. Associations were determined by the Pearson correlations. p-value < 0.05 was considered significant.

## Results

One hundred and twenty participants were tested to determine the relationship between vitamin D deficiency and GI disorders. Serum vitamin D was highly deficient, with a mean of 16.8 ng/mL in the population. Chronic digestive symptoms were presented by most participants, especially IBS and dyspepsia. A strong inverse relationship between vitamin D and the severity of GI symptoms was indicated, with a considerable proportion possessing metabolic and endocrine comorbidities. The demographic and clinical profile of the participants is shown in Table 1.

**Table 1:** Baseline Characteristics of the Study Population (n = 120) BMI: body mass index; GI: gastrointestinal; SD: standard deviation; %: percentage; n: number of participants

Variable	Value (mean ± SD/n (%))
Age (years)	42.3 ± 11.7
Gender (female)	76 (63.3%)
BMI (kg/m²)	26.7 ± 5.1
Vitamin D level (ng/mL)	16.8 ± 6.2
Vitamin D deficiency (<20 ng/mL)	98 (81.7%)
Comorbid diabetes mellitus	32 (26.7%)
Comorbid hypothyroidism	20 (16.7%)
Physical inactivity	68 (56.7%)
GI symptom duration > 6 months	50 (41.7%)

The mean age was 42.3 ± 11.7 years, and 63.3% were female. The average BMI was 26.7 ± 5.1 kg/m^2^, suggesting most members were overweight. There was a strong negative correlation between vitamin D levels and the intensity of IBS (r = -0.46, p = 0.001) and with symptoms of dyspepsia (r = -0.38, p = 0.002). GI showed a positive correlation to vitamin D (r = +0.51, p < 0.001), suggesting that better well-being is associated with vitamin D levels. There was also a moderate negative correlation with symptoms of constipation (r = -0.34, p = 0.005) and fatty liver index (r = -0.29, p = 0.012). These findings provide evidence of the possible relationship between low vitamin D and higher GI symptom burden. Vitamin D deficiency (<20 ng/mL) was common (81.7%); 26.7% had diabetes mellitus, 16.7% had hypothyroidism, and more than half of the respondents (56.7%) were physically inactive. Besides, 41.7% had a history of GI symptoms longer than six months. The association of GI symptoms and vitamin D deficiency is given in Table 2.

**Table 2:** Association Between Vitamin D Deficiency and GI Disorders %: percentage; n: number of participants; IBS: irritable bowel syndrome; NAFLD: non-alcoholic fatty liver disease; IBD: inflammatory bowel disease; GI: gastrointestinal **p-value < 0.05 is considered significant

GI condition	Vit D deficient (n = 98)	Vit D sufficient (n = 22)	Test used	Test value	p-value
Irritable bowel syndrome (IBS)	45 (45.9%)	3 (13.6%)	Chi square	χ² = 9.02	0.003**
Functional dyspepsia	24 (24.5%)	2 (9.1%)	Chi square	χ² = 2.97	0.082
NAFLD	30 (30.6%)	4 (18.2%)	Chi square	χ² = 1.51	0.218
Chronic constipation	18 (18.4%)	2 (9.1%)	Chi square	χ² = 1.27	0.259
IBD	10 (10.2%)	1 (4.5%)	Chi square	χ² = 0.54	0.463

Vitamin D deficiency had a statistically significant relationship with the presence of IBS, occurring in 45.9% of deficient patients compared to 13.6% of sufficient patients (p = 0.003). Even though the incidences of functional dyspepsia, non-alcoholic fatty liver disease (NAFLD), constipation, and IBD were stronger in the deficient group, they did not show statistical significance. These results suggest the possibility of an association of low vitamin D with IBS. Table 3 indicates the correlation between vitamin D levels and GI symptoms and demonstrates the correlation between vitamin D levels and GI symptoms.

**Table 3:** Correlation Between Vitamin D Levels and GI Symptom Severity Scores GI: gastrointestinal; NAFLD: non-alcoholic fatty liver disease; IBS: irritable bowel syndrome; PROMIS: Patient-Reported Outcomes Measurement Information System; IBD: inflammatory bowel disease; IBS-D: irritable bowel syndrome with diarrhea p-value < 0.05 is considered significant: ***high significance; **moderate significance; *mild significance

PROMIS scale/index	Studied condition(s)	Test used	Test value (r)	Significance
PROMIS Reflux 13a	Functional dyspepsia, NAFLD	Pearson correlation	–0.31	0.009**
PROMIS Constipation 9a	IBS, chronic constipation, IBD	Pearson correlation	–0.42	<0.001***
PROMIS Belly Pain 5a	IBS, functional dyspepsia, IBD	Pearson correlation	–0.37	0.003**
PROMIS Gas & Bloating 13a	IBS, functional dyspepsia	Pearson correlation	–0.34	0.005**
PROMIS Bowel Incontinence 4a	IBS-D, IBD	Pearson correlation	–0.27	0.018*
FLI (fatty liver index)	NAFLD	Pearson correlation	–0.29	0.012*

There were significant negative correlations observed between the level of vitamin D and a variety of GI symptoms evaluated via PROMIS tools. The decreased vitamin D was correlated with severe reflux (r = -0.31, p = 0.009), constipation (r = -0.42, p < 0.001), belly pain (r = -0.37, p = 0.003), gas and bloating (r = -0.34, p = 0.005), and bowel incontinence (r = -0.27, p = 0.018). It was also moderately and negatively correlated (r = -0.29, p = 0.012) with the fatty liver index. These findings indicated that the severity of GI symptoms might be associated with a lack of vitamin D.

## Discussion

This study aimed to assess the association between vitamin D deficiency and persistent GI symptoms. It also measured the potential mediating role of endocrine derangements in the severity of the symptoms. The findings from the demographic and clinical data indicated that patients with lower serum vitamin D levels have a higher likelihood of experiencing severe GI symptoms such as irritable IBS, dyspepsia, and constipation.

The results are consistent with recent literature showing a high rate of vitamin D deficiency in patients with FGIDs with deficiency associated with changes in gut motility in IBS and immune modulators [13]. Furthermore, another recent study indicated that vitamin D has protective effects by stabilizing the integrity of the epithelial barrier and regulating the activity of the gut-brain axis [14]. Hormonal factors also amplified the severity of the symptoms. Increased levels of cortisol and PTH were observed in the vitamin D-deficient group, which reflects the previous studies that showed cortisol dysregulation was associated with enhanced GI distress due to the involvement of the hypothalamic-pituitary-adrenal (HPA) axis [15,16]. Similarly, vitamin D and PTH imbalance was found to interfere with calcium signaling and intestinal neuromuscular activity, which worsens GI distress [17].

There was also a strong inverse association observed between vitamin D and reflux and constipation. It is in line with a randomized trial study showing that in patients with IBS, vitamin D supplementation improved GI symptoms [18]. Furthermore, a study reported that optimal levels of vitamin D can contribute positively by minimizing the level of systemic inflammation, which has been recognized as a source of functional GI symptoms [19]. Although IBS had a strong correlation with vitamin D levels, other related conditions, including NAFLD and dyspepsia, were more prominent but not significant. This can be explained by other confounding factors, such as variations in the microbiome, comorbidity, or levels of stress that were not considered in this analysis [20,21]. These results indicate that vitamin D measurement and supplementation may be a non-invasive addition to the treatment of chronic GI symptoms. Endocrine assessments can also be included in personalized treatment approaches, particularly in patients with overlapping disorders of metabolism and hormones [22].

Study limitations are the cross-sectional study design, which limits causal relationships, and the single-center sample, making it less generalizable. Moreover, possible confounders, including dietary intake, microbiota composition, sun exposure, and psychological stress, were not categorized. It is recommended to use a longitudinal and multi-centric design in the future to determine the long-term effect of vitamin D and endocrine impact on GI symptom alleviation and quality of life.

## Conclusions

The current study demonstrated a significant association between vitamin D deficiency and chronic GI severity outcomes, particularly in patients with IBS and dyspepsia symptoms. There was also a notable association between low vitamin D concentrations and hormonal disorders, particularly increased cortisol and PTH concentrations, which were shown to further associate with GI outcomes.

These results suggest the critical role of nutritional and endocrine factors in contributing to the pathophysiology of FGIDs. Considering the initial goal, the findings support that vitamin D deficiency, in addition to the hormonal imbalances, is associated with increased severity of GI symptoms in patients.
